# Inflammatory modulation of exercise salience: using hormesis to return to a healthy lifestyle

**DOI:** 10.1186/1743-7075-7-87

**Published:** 2010-12-09

**Authors:** Alistair V Nunn, Geoffrey W Guy, James S Brodie, Jimmy D Bell

**Affiliations:** 1 Metabolic and Molecular Imaging Group, MRC Clinical Sciences Centre, Hammersmith Hospital, Imperial College London, Du Cane Road, London W12 OHS, UK; 2 GW pharmaceuticals, Porton Down, Salisbury, Wiltshire SP4 0JQ, UK

## Abstract

Most of the human population in the western world has access to unlimited calories and leads an increasingly sedentary lifestyle. The propensity to undertake voluntary exercise or indulge in spontaneous physical exercise, which might be termed "exercise salience", is drawing increased scientific attention. Despite its genetic aspects, this complex behaviour is clearly modulated by the environment and influenced by physiological states. Inflammation is often overlooked as one of these conditions even though it is known to induce a state of reduced mobility. Chronic subclinical inflammation is associated with the metabolic syndrome; a largely lifestyle-induced disease which can lead to decreased exercise salience. The result is a vicious cycle that increases oxidative stress and reduces metabolic flexibility and perpetuates the disease state. In contrast, hormetic stimuli can induce an anti-inflammatory phenotype, thereby enhancing exercise salience, leading to greater biological fitness and improved functional longevity. One general consequence of hormesis is upregulation of mitochondrial function and resistance to oxidative stress. Examples of hormetic factors include calorie restriction, extreme environmental temperatures, physical activity and polyphenols. The hormetic modulation of inflammation, and thus, exercise salience, may help to explain the highly heterogeneous expression of voluntary exercise behaviour and therefore body composition phenotypes of humans living in similar obesogenic environments.

## Introduction

Ancient man was a hunter-gatherer, often travelling long distances to find food, avoid threats and seek shelter. In contrast many modern western societies have transformed their surroundings in order to minimise (or even eliminate) environmental threats and stresses that our ancestors were exposed to, including food and water shortages, predation, infections, extremes of temperature and the need to carry out regular physical activity. Moreover, the modern western environment now contains almost unlimited supplies of foods containing high level of saturated fats, salt and refined sugars. This also appears to be coupled with a reduction in the consumption of plant products including fruits, nuts and vegetables. The result of this obesogenic environment is a burgeoning rise in lifestyle-induced diseases that are generally associated with energy imbalance, abnormal fat deposition and inflammation. The links between obesity, the metabolic syndrome, diabetes, vascular disease and even cancer, and a sedentary lifestyle, are now all too clear.

One field of emerging interest in obesity research is the study of what motivates people to undertake voluntary physical activity, or display spontaneous physical activity. Indeed, understanding its biological basis is an important research goal [1]. This behaviour could be described by a cognitive state that we have termed "'exercise salience". It is therefore essential to determine the factors, genetic and environmental, that regulate a person's exercise salience, in particular to understand what suppresses it. Here, we propose that one such factor is lifestyle-induced chronic subclinical inflammation; markers of the metabolic syndrome include raised hs-CRP (high sensitivity C-reactive protein) and reduced adiponectin [2]. The rationale for this is the well described behavioural change associated with injury and infection, which is commonly known as "inflammatory-induced sickness behaviour" [3].

We therefore propose that exercise salience, the motivation to undertake physical activity, is modulated by the inflammatory status of an animal, decreasing in an inflammatory phenotype, including the metabolic syndrome and increasing in an anti-inflammatory "healthy" phenotype. The type of phenotype may well be determined by the degree of hormesis, as metabolic stressors, such as exercise, plant polyphenols and calorie restriction tend to induce an anti-inflammatory phenotype. In effect, without constant environmental hormetic priming, and in the presence of unremitting caloric surplus, many modern humans may tip into a chronic subclinical inflammatory zone, where physical activity becomes less and less palatable, both physically, and psychologically. They then enter a vicious cycle which reinforces the development of a sedentary phenotype and the metabolic syndrome. Thus humankind may be suffering from an "intelligence-driven health paradox", as intelligence has enabled us to remove the very hormetic factors that have been responsible for ensuring our biological fitness.

## Inflammation may suppress exercise salience

Reduced physical activity is strongly associated with increased obesity [4]. Furthermore, it appears that spontaneous physical activity correlates with free living habitual physical activity - and could help to explain the diversity in body adiposity observed in humans inhabiting similar obesogenic environments [5]. The precise biological basis for spontaneous physical activity is far from understood, and although there is a degree of heritability, a complex trait like this probably involves many genes and displays a multifaceted interaction with body composition [6-8].

In light of this, it is interesting that human studies increasingly support the role of inflammation in the modulation of a number of cognitive executive functions [9]. "Inflammatory-induced sickness behaviour" has been recognized for many years and is associated with a behavioural response to ensure that an organism can recover as quickly as possible in response to injury and/or infection. Given the opportunity, an injured animal would seek to reduce all physical activities suggesting that the immune system appears to "subjugate the brain", probably by way of inflammatory cytokines [10]. For example direct injection of cytokines, or injection of agents, such as LPS (lipopolysaccharide) that induce cytokine release, result in depressive symptoms, anxiety and invoke fatigue and reduced movement [11]. Critically, some anti-inflammatory compounds can block/reverse many of these changes [12]. Moreover, data suggest that raised levels of hs-CRP (a marker of the inflammatory acute phase response) are strongly related to major depressive disorder and support clinical observations that immunotherapy often induces depression [13].

There is thus a paradigm for inflammation to suppress exercise salience. Studies continue to suggest that a lack of physical activity is associated with reduced cognitive executive functions [14,15], with depression being recognised as a component of the metabolic syndrome [16,17]. Moreover, exercise, which is strongly anti-inflammatory [18] is well known to have anti-depressive qualities. In fact, research now supports the notion that not only is cardio-respiratory fitness inversely related to the presence of the metabolic syndrome, but even if markers of the metabolic syndrome are present, their risk is significantly reduced by increased respiratory fitness [19,20].

## Can exercise salience be enhanced by suppression of inflammation?

It is now well established that many medicinal plant compounds have anti-inflammatory properties, and that calorie restriction and physical activity tend to result in a phenotype that resists oxidative stress, and thus chronic inflammation [21,22]. One striking observation is that both calorie restriction and endurance exercise have strong anti-inflammatory effects in adipose tissue, which can result in reduced insulin resistance [23]. Resveratrol, a plant polyphenol with calorie restriction mimicking effects, has also been shown to have anti-inflammatory effects in adipose tissue [24]. This is an important finding given that inflammation in adipose tissue is now recognised as one of the major factors associated with the development of the metabolic syndrome [25].

In fruit flies, resveratrol can lead to an increased propensity to undertake physical activity and appears to be associated with increased sirtuin activity [26]. Daily feeding of quercetin to mice increases mitochondrial biogenesis in both muscle and brain, improves exercise tolerance and stimulates voluntary wheel running [27]. Similarly, feeding green tea to mice can increase exercise endurance [28], while quercetin can both increase VO_2 _max and endurance in untrained human volunteers [29]. Resveratrol can also induce mitochondrial biogenesis in human endothelial cells *in vitro *[30]. Other studies have shown that although resveratrol fed to mice can increase mitochondrial biogenesis and exercise endurance, it is not associated with increased locomotor activity [31]. However, overall, there is clear evidence that some anti-inflammatory polyphenols can indeed induce exercise salience in animals, and in common with these models, there is now evidence that they might possibly improve exercise tolerance in humans.

Calorie restriction generally refers to the practice of restricting the daily calorie intake that an animal or human would normally eat *ad libitum*, but maintaining sufficient nutrition to keep them healthy. Alternate day fasting has also been shown to display many of the same benefits, but the data in humans is less clear because the few trials that have been undertaken are relatively short. Moreover, alternate day fasting does not always lead to weight loss, suggesting a heterogeneous response [32]. In relation to exercise salience, calorie restriction is known to induce increased voluntary wheel running in rodents, which is often associated with reduced appetite and is thought to represent a "flee from famine" response. Although acute starvation induces both behavioural and metabolic adaptations to save energy, prolonged partial starvation accompanied with weight loss can induce a switch to foraging behaviour and an increased propensity to move. This may well be species dependent to some degree (e.g. hibernating versus migrating animals), as well as partially sex dependent: female rats appear to display increased running behaviour sooner than male rats when calorie restricted [33]. Importantly, it may also be contextual in relation to food availability. If normal weight rats are fed once a day, rather than allowing them to feed little and often as they normally do, they may develop anorexia and hyperactivity and can literally run themselves to death if a wheel is available. In the absence of a wheel this extreme behaviour does not appear to take place. However, there is heterogeneity in the expression of this behaviour, which may represent potential genetic differences [34]. This heterogeneity is also seen in non-human primates: in one study where rhesus monkeys underwent calorie restriction of 30% for 6 years, there was a clear increase in physical activity compared to *ad libitum *fed animals [35]. In contrast, in another study also involving rhesus monkeys calorie restricted by 30%, there was no difference in physical activity levels compared with controls after 30 months [36]. Thus, the predilection to either stay put and conserve energy, or get up and seek food are both potential survival strategies dictated by the environment and modulated by the genetic background of each individual.

Similar to animals, calorie restriction does improve most metabolic markers in humans and may improve "healthspan", and quite possibly, lifespan [37]. Calorie restriction also increases muscle mitochondrial biogenesis in humans [38]. As indicated, alternate day fasting may also confer some of the same benefits as daily calorie restriction. In a small short term study, obese subjects were exposed to alternate day 25% calorie restriction for 8 weeks. Despite achieving an average weight loss of 5-6%, the subjects showed no reduction in their daily physical activity levels [39]. However, 50% calorie restriction (effectively semi-starvation) of lean human males greatly reduced spontaneous physical activity over a 24 week period [40]. Likewise, calorie restriction of 25% for 24 weeks in healthy, but overweight and mainly sedentary subjects also resulted in the induction of classical metabolic adaptation to reduce energy expenditure, including reduced voluntary physical activity. In contrast, another group in this study who underwent 12.5% calorie restriction plus a 12.5% increase in physical activity did not show any metabolic adaptation to calorie restriction [41]. Other than genetics, explanations for the heterogeneity in exercise salience may well include current energy balance (fat stores), cognitive assessment of the likelihood of getting another meal, the polyphenolic content of the diet, as well as the amount of physical activity.

Thus it appears that at least potentially three anti-inflammatory factors, calorie restriction, plant polyphenols and even exercise itself may engender exercise salience. With regards physical activity, running is well known to be rewarding and addictive in humans, and certainly has anti-depressive qualities [42]. This is supported by the fact that resistance training in otherwise sedentary children can also lead to an increase in spontaneous physical activity [43].

## The anti-inflammatory effects of hormesis and the mitochondrial metabolic engine

Calorie restriction, plant polyphenols and physical activity are metabolic stressors which display a property known as "hormesis". Hormesis describes a biphasic dose response whereby a small dose of stress stimulates resistance to that stress and improves biological fitness, while too much stress induces damage and inhibits function. "Hormetins", in physiological terms, can be described as any condition that results in upregulation of the cellular stress response system, in particular, any which invokes metabolic and oxidative stress [44], of which exercise is one of the best described [45]. Hormetins also include polyphenols and calorie restriction [46], as well as temperature stress [47].

Throughout evolution, most species, due to predation, disease, shortage of food, accidents and other environmental factors, rarely lived beyond 50% of their maximal possible life span [48]. Life developed in a stressful environment and evolved the necessary mechanisms to utilise these "stresses" to optimise function and at the same time minimise their detrimental effects. It can therefore be said that life evolved in a "hormetic zone", where stress ensured optimal biological fitness [49]. Although there is little direct evidence, other than the well known benefits of exercise, it is thought that that hormesis could potentially be of great benefit to humans [50-52].

Key in hormesis is the adaptive response to oxidative stress involving both the plasma membrane and the mitochondrion, and modulation of multiple signalling pathways. Stressful stimuli, which usually result in increased energy demand, tend to activate similar intracellular programmes that upregulate anti-oxidant systems and usually, mitochondrial biogenesis. Critical in this are factors such as nuclear factor erythroid 2-related factor 1/2 (Nrf-1/2), PPAR γ coactivator 1 (PGC-1), forkhead transcription factors (FOXO), sirtuins, AMP kinase (AMPK), mitochondrial transcription factor A (Tfam) and heat shock proteins (HSPs) [53-55]. An important part of this process may well be mitochondrial-nucleus retrograde signalling involving redox. Indeed, this process may be very ancient and reflect the importance of the mitochondrion in detecting energy flux, and via alterations in redox, instigate appropriate epigenetic changes to ensure optimum cellular phenotype; primary in this is maintaining efficient mitochondrial function and the ability to burn fatty acids [56].

Extreme longevity (as displayed by primates, especially humans), appears to be associated with rapid evolution of mitochondrial proteins involved in oxidative phosphorylation, resulting in increased uncoupling, ATP/free radical ratios and metabolic rates [57]. Of the 1,500 or so mitochondrial genes, only 13 polypeptide encoding genes remain in the mitochondrial genome - the rest being encoded in the nuclear genome. This appears to be common across most kingdoms, suggesting these particular genes are critical. One possible explanation is that the high rate of mitochondrial gene mutation may be important to ensure optimal biological fitness. It also suggests that mitochondrial dysfunction may underlie many common human diseases. In fact, many different mitochondrial haplotypes exist in humanity, implying significant adaptation to different environments. For instance, tight coupling where thermogenesis is less important, such as in hot countries, might result in high ATP phenotypes, where as in cold countries, increased uncoupling may help in generating heat (for a comprehensive review on the subject, the reader is directed to Wallace 2008) [58]. Research indicates that some haplotypes may be better protected against type II diabetes [59], while others are associated with extreme longevity [60]. Of particular relevance is the role of PGC-1α in adaptive thermogenesis; this coactivator is key in upregulating mitochondrial biogenesis and uncoupling to generate heat [61]. It has been suggested that it may well play a key role in adaptation of some ethnic groups to the cold, which may protect them to some degree against type II diabetes [62].

An important component of hormesis is the potential to upregulate total mitochondrial function and thus the ability to generate ATP from the proton gradient. An emerging concept that ties in with this is the idea that the resting metabolic rate (RMR) reflects an animal's "metabolic engine" size and capacity to do work, and that this may determine its behaviour and fitness [63]. For instance a bigger metabolic engine enables increased foraging activity, aggressiveness or courtship and importantly, the ability to grow and store energy. The downside to this is that the animal will have a higher energy consumption at rest - so potentially putting it at a disadvantage when food availability is very low. If the concept of hormesis is combined with this, it suggests that environmental challenges will act to increase the metabolic engine size of an animal, but that this must necessarily be modulated to local situations. Thus, from the viewpoint of calorie restriction, mitochondrial biogenesis increases the capacity of the metabolic engine, but the restricted calories may well slow the engine "tickover" to reduce energy output. However, it prepares the animal to be competitive when food is available. Temperature extremes, exercise and polyphenols would exert an immediate increase in metabolic engine size to ensure survival.

In this context, it is therefore likely that inflammation must play a role in controlling both physiological function and behaviour, which is intimately related to mitochondrial function. In order to allocate resources towards repair and fighting infection, inflammation must therefore suppress unnecessary energy usage and engender an anti-pathogenic cellular environment. This may involve reduced oxidative phosphorylation (and a shift towards glycolysis), the development of insulin resistance and a shift to a more oxidising intracellular environment. This would have to be combined with reduced physical activity to prevent excessive energy use and to allow repair of damaged organs/limbs.

## Plants, mitochondria and exercise salience - a very old partnership?

As polyphenols are part of plant defence/stress signalling it has been suggested that animals have adopted these compounds as xenohormetic signals. This may provide them with information about changes in the environment [64]. This coevolution and interaction may be very ancient. In effect, the development of a potent stress resistance system by plants may have been adopted by animals [65], suggesting that hormetic effect of plants on animals has been influencing exercise salience throughout their evolution.

Plants and animals both have mitochondria, and display many similarities, for instance, mitochondrial involvement during apoptosis [66]. It is believed that mitochondria were originally bacterial endosymbioants that gave rise to two kingdoms when they became incorporated in nucleated cells: plants and animals. In plants, phenolics and polyphenols play important roles in ensuring plants are resistant to different types of stress [67]. In fact, it has been recently suggested that mitochondria were a pre-requisite of complex life as bioenergetically they allowed eukaryotic cells to contain and express many more genes than prokaryotes [68].

One important signalling mechanism involves ROS [69] and several well described plant stress signal molecules, such as salicylic acid, directly modulate mitochondrial function (and thus, ROS) by uncoupling [70]. Data from animal cells also show that not only are ROS important in directly modulating mitochondrial function, but so are calcium and nitric oxide (NO) and other reactive nitrogen species [71,72]. It appears that NO is just as important in plants as it is animals in relation to signalling and defence [73]. Furthermore, many plant flavonoids directly modulate the mitochondrial calcium uniporter [74]. Hence, polyphenolic modulation of key cellular signalling molecules that can both modulate and be modulated by mitochondria is a trait that appears to cross kingdoms.

Tapia (2006) suggested that many polyphenols stimulate mitochondrial biogenesis by inducing sublethal mitochondrial stress and production of ROS, resulting in a better protected phenotype [46]. Certainly, resveratrol, quercetin and epigallocatechin gallate (EGCG) can induced mitochondrial biogenesis, which could be linked to increased muscle performance and endurance [75-77]. Critically, quercetin, EGCG and berberine (another bioactive polyphenol), have now been found to preferentially concentrate in mitochondria [78-80]. This suggests that many plant polyphenols can directly alter mitochondrial function and biogenesis, cell cycling, proliferation and death. The precise effect would depend on the mix of polyphenols and the type of cell, but there might be a more generalised biphasic dose effect. It could be said that plant-induced hormesis, which tends to have an anti-inflammatory effect in many animals, would also provide a xenohormetic signal to stimulate movement as a survival strategy.

## Tipping in and out of the metabolic syndrome; the role of exercise salience and hormesis

The above suggests that hormetins play a crucial role in reversing the metabolic syndrome. We have previously suggested that the metabolic syndrome may have its origins in thriftiness, insulin resistance and the redox signalling system [81]. The underlying premise is that thriftiness results from an evolutionarily-driven propensity to minimise energy expenditure, which has to be balanced with the need to resist the oxidative stress from cellular signalling and pathogen resistance; this gave rise to "redox-thriftiness". In effect, mitochondria may be able to both amplify membrane-derived redox growth signals as well as negatively regulate them, resulting in an increased ATP/ROS ratio. In times of relative plenty, anabolic drive and redox-thriftiness leads to mild insulin resistance in some tissues, which has the effect of both protecting the individual cell from excessive growth/inflammatory stress, while ensuring energy is channelled to the brain, the immune system, and for storage. In turn, fine tuning of redox thriftiness is achieved by hormetic signals that stimulate mitochondrial biogenesis and resistance to oxidative stress, which improves metabolic flexibility and suppresses excessive inflammation. Hence, in a non-hormetic environment with excessive calories, the protective nature of this system may lead to escalating insulin resistance and rising oxidative stress due to metabolic inflexibility and mitochondrial overload. Genetically and environmentally determined mitochondrial function may then define a "tipping point" where protective insulin resistance tips over to inflammatory insulin resistance, and loss of adipose plasticity and capacity. In effect, once on the wrong side of this tipping point, an organism simply cannot deal with excessive calories, which then end up getting deposited ectopically - so perpetuating the cycle.

A critical component of this loss of adipose plasticity appears to be inflammation. In adult animals, although adipogenesis can occur, most fat storage probably occurs via adipocyte hypertrophy. However, too much fat can initiate adipocyte "overload distress", especially in intra-abdominal adipose tissue (IAAT, otherwise known as "visceral" fat), which leads to activation of the immune system and the acute phase response (APR); the distressed adipose tissue acts as a focus for inflammatory macrophages [82,83]. Importantly, it seems that this process appears to drive preadipocytes towards an inflammatory macrophage phenotype, rather than maturing into fat storing cells - so further amplifying the inflammatory milieu, thus losing storage capacity and enhancing ectopic fat deposition [84].

As might be expected from a loss of metabolic flexibility, one of the earliest components of the metabolic syndrome appears to be the development of excessive fat in liver, which correlates well with the development of excessive IAAT and insulin resistance [85,86]. Indeed, liver fat may be a better marker than abnormal IAAT for the metabolic syndrome [87]. However, there is a large variability in liver fat levels in relation to pathology. One explanation is that the liver may play an important thermogenic function as part of an adaptation to utilise fatty acids [88]. Another is suggested by the observation that if rats are fed a high fat diet, simultaneous exercise reduces liver fat. However, intermittent, rather than continuous exercise (amounting to the same total energy expenditure), may be far more effective at reducing this fat depot [89]. Hence, not only does exercise reduce liver fat, but short bursts may be better; this would be line with hormesis - as chronic exercise may be expected to induce adaptive changes.

For most animals, including humans, lack of hormetic stimuli in the presence of excessive calories will not only lead to obesity, but to an aberrant fat distribution, which underlies the basis of the metabolic syndrome. Under normal circumstances, fat depots do display considerable plasticity. For instance, in response to a high fat diet, IAAT adipocytes in mice initially become hypertrophic and undergo apoptosis and attract inflammatory macrophages. However, after a period of 12-20 weeks, the IAAT remodels itself, reducing in size with smaller adipocytes - which may be related to an increase in anti-inflammatory cytokines and improved insulin sensitivity. In contrast, subcutaneous adipose tissue (SCAT) hardly changes. Beyond this time point, if the high fat diet is continued, liver fat deposition increases. This may represent a plastic change in IAAT, which first absorbs the extra fat, but then becomes resistant to further increases in size [90]. This would support the concept that it has a critical size threshold, beyond which is becomes pathological [91] and the observation that IAAT size can determine life expectancy [92].

In the context of evolution, it is possible that these adipose-derived inflammatory signals may be entirely physiological. For instance, a mild inflammatory signal that induces a sedentary behaviour, which in turn is associated with mild muscle insulin resistance, may ensure deposition of fat in SCAT. In wild animals, this might correspond to a feast period during a time of plenty (e.g. after the rains or in the summer). Support for this comes from the observation that mice exposed to a human western diet very quickly show reduced locomotor activity [93] - although the opposite can occur in mice bred for their high voluntary wheel running activity: the increase in fats appears to drive/support increased activity [94]. Hence, in a continuous obesogenic environment with reduced hormesis, an animal, without any evidence of physical injury, may cross a tipping point and display inflammatory-induced sickness behaviour, with reduced exercise salience. In effect, the metabolic flexibility of an animal will dictate the degree of resistance to transiting to the inflammatory zone.

**Figure 1:**
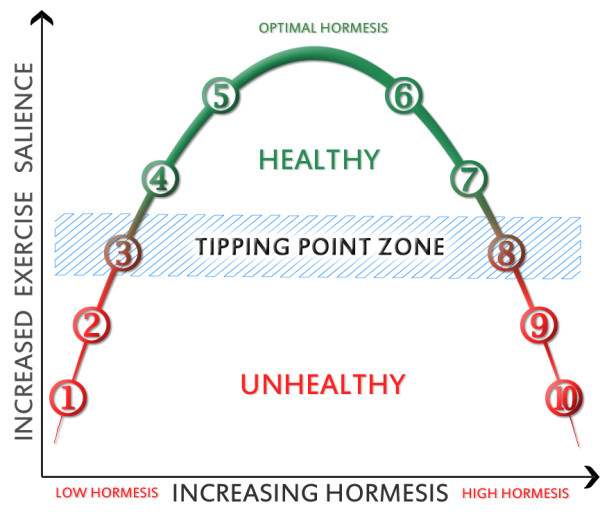
**The theoretical relationship between the biphasic hormetic curve and exercise salience**. Regular hormetic stressors result in better mitochondrial function and increased resistance to oxidative stress, which translates into reduced inflammatory tone, improved metabolic flexibility and higher exercise salience. Without regular hormetic stressors and/or with chronic inflammation (e.g. from an injury or infection), an animal may cross a tipping point and remain in an inflammatory state due to a feed forward loop (1 and 2). In times of plenty (but with some stressors), it may exist in zone 3, where optimal energy storage occurs (mild inflammation induces insulin resistance) - but it is still relatively metabolic flexible. Beyond this, regular hormetic stressors would act to induce further exercise salience (zones 4 and 5). However, as hormetic stressors increased, damage would result in a gradual decrease in function and reducing exercise salience (6 and 7) until the animal had to slow down to recover (8). If excessive stress continued, it might develop chronic inflammation, and eventually succumb (9 and 10). The ability to resist transiting zones may be associated with an epigenetic shifting of the tipping point. In effect, an animal may adapt over time (or be preprogrammed from the preceding generations). In a non-hormetic environment, this would reinforce the inflammatory cycle, while in a hormetic environment, the anti-inflammatory cycle will predominate. Much of the Western society appears to reside in zone 2 due to a lack of hormesis and an excess of calories. Key to zones: 1 = inflammatory induced sickness behaviour; 2 = subclinical inflammation; 3 = remain sedentary and store food; 4 = active and seek food; 5 = migratory; 6 = late migratory; 7 = stress induced damage; 8 = sedentary recovery zone; 9 = inflammatory induced sickness behaviour; 10 = failure of systems and death.

In contrast, continued hormetic stimuli may not only increase the resistance to transiting to the inflammatory zone, but may tip an organism towards a non-inflammatory phenotype. In this regard, the effect of exercise is particularly interesting, as regular exercise rapidly and preferentially depletes abdominal obesity [95]. This may also initiate a feed forward loop, but unlike the inflammatory phenotype, this phenotype may have a continual urge to increase/maintain physical activity (high exercise salience). At a mild level, this ensures the animal continues to move to look for new food sources, or flee from stressful environment. At its extreme, as has been previously suggested [96], it could represent an anorexic "flee from famine" phenotype - in humans this may be represented by those addicted to exercise, while in the wild it might be represented by a migratory phenotype in some species. It would thus be triggered by environmental stimuli such as famine, or possibly, even drought or factors that might induce an animal to move from danger (i.e. enforced physical activity, such as unacceptable levels of predation or competition). Moreover, through plant polyphenols, animals would also be able to obtain further environmental early warning as the plants become stressed. As hormesis is biphasic, too much stress would clearly start to become detrimental and eventually induce inflammation and slow the animal down. A theoretical relationship between hormesis and exercise salience is depicted in Figure 1.

## Exercise salience in the modern world; revving up the metabolic engine

As we have described, reduced exercise salience may be closely linked to increased inflammation. In an obesogenic environment this leads to increasing prevalence and severity of the metabolic syndrome. At the molecular level this arises from insufficient redox stimuli and reduced mitochondrial functioning and decreased resistance to oxidative stress. Without redox challenges there is reduced adaptation and metabolic fitness and the metabolic engine capacity decreases. Once a subject crosses the inflammatory tipping point it may be more difficult to return leading to a vicious cycle of further oxidative stress, abnormal fat distribution and reduced exercise salience. This may not only affect an individual's immediate phenotype, but via epigenetics (and parental behavioural reinforcement), the chances of future generations.

So what is to be done to maximise the health in a modern society? We know that hormetic stimuli can induce a phenotype that actively seeks and stores energy to improve its ability to survive. This stress-energy seeking paradigm may be a fundamental to much of life. Clearly, the way forward for humanity (and his crops and food animals) would be to increase hormetic stimuli. For mankind, exercise is probably one of the strongest mechanisms to achieve this, as reduced physical activity levels and low general fitness are strongly associated with the metabolic syndrome - even in adolescents [97]. Fasting and calorie restriction is another alternative; even alternate day fasting may provide many of the same benefits as sustained calorie restriction [98]. We probably need a combination of factors to both increase the metabolic engine size and raise the tickover.

Fatty acids, especially unsaturated fatty acids, may also help by inducing mitochondrial ROS production, and thus, mitochondrial biogenesis; they could be said to display hormetic properties [81]. Interestingly, some migratory birds eat large amounts of n-3 polyunsaturated fatty acid (PUFA) containing foods before migration, which has been described as a kind of performance "doping" to enhance muscle efficiency on very long flights [99]. We suggest this might also be a kind of hormetic signal from the environment. This would in part help to explain why some PUFAs are associated with better health and may well be enhancing exercise salience.

Similarly, the role of polyphenols in our daily diet should be considered. The benefits of a Mediterranean diet are well known, but data on individual polyphenols is also very informative. Daily feeding of quercetin to mice has been shown to not only improve muscle mitochondrial biogenesis and endurance, but also increase voluntary wheel running [100], while resveratrol can improve lipopolysaccharide (LPS)-induced working memory deficit in aged mice [101]. Furthermore, reverting to a Palaeolithic diet can improve many metabolic parameters even in apparently healthy but sedentary human subjects. The important factor may be the increase in plant nutrients derived from nuts and berries, and the increase in the unsaturated to saturated fat ratio [102].

It is also possible that different ethnic groups may have evolved varying levels and/or patterns of hormetic dependencies - possibly reflecting different mitochondrial haplotypes. For instance, cold adapted groups may be better able to resist developing type II diabetes [62]. This may partly explain an emerging type II diabetes "red-zone", which broadly follows the Tropic of Cancer and therefore a warmer climate, which involves countries in North Africa, through the Middle East, to India and China. The Arab ethnicity appears to be particular affected [103,104]. In these countries it is possible that rapid economic growth has enabled a constant supply of water, the use of air-conditioning and the adoption of a western diet: drought and heat may have been important daily hormetic stimuli in these regions (both directly, and indirectly via food plants and animals), which have now been removed. This pattern would be clearly different for those adapted to colder climates at higher latitudes. Equally, this suggests that although many people in the developed nations may eat plenty of fruit and vegetables, they may not be getting the full beneficial effect as most of this food is grown in highly controlled environments, with little stress and may therefore contain minimal hormetic stress signals. A sentiment recently echoed by Hooper and colleagues [105]. An example of this is that climatically stressed grapes tend to contain more resveratrol [106]. It may be time to revisit eating locally grown seasonal vegetables and fruits.

In conclusion, the decline in hormetic stimuli in our daily life may be leading to increased systemic sub-clinical inflammatory tone, decreased metabolic flexibility and suppression of exercise salience. All of which translate into a significant increase in chronic diseases. Whether we like it or not, a long and healthy life needs to include regular exposure to occasional doses of environmental stressors, including fasting, natural temperature changes, polyphenols and exercise. Although human intelligence has enabled us to remove most stressors from the environment, common sense may be required to re-introduce some of them.

## Competing interests

The authors declare that they have no competing interests.

## Authors' contributions

AVN, with contributions from JDB and GWG, developed the original concept and wrote the manuscript. JSB contributed in the preparation and critical review of manuscript. All authors read and approved the final manuscript.
